# Genetic Selection to Enhance Animal Welfare Using Meat Inspection Data from Slaughter Plants

**DOI:** 10.3390/ani8020016

**Published:** 2018-01-24

**Authors:** Pramod K. Mathur, Roos Vogelzang, Herman A. Mulder, Egbert F. Knol

**Affiliations:** 1 Topigs Norsvin BV, P.O. Box 43, Beuningen 6640 AA, The Netherlands; Roos.Vogelzang@topigsnorsvin.com (R.V.); egbert.knol@topigsnorsvin.com (E.F.K.); 2 Wageningen University & Research Animal Breeding and Genomics, P.O. Box 338, Wageningen 6700 AH, The Netherlands; han.mulder@wur.nl

**Keywords:** welfare, disease, meat inspection, remark, genetic selection, pigs

## Abstract

**Simple Summary:**

Analysis of a large volume of meat inspection data suggests availability of genetic variation for most common indicators of poor animal welfare. This genetic variation can be used to select pigs that have the potential to resist common infections and other unfavorable welfare conditions. Genetic selection can be a tool in addition to farm management in reducing the risk of diseases, thereby reducing pain and suffering of animals. In general, the slaughter remarks have small but favorable genetic relationships with finishing and carcass quality traits. Therefore, it is possible to enhance animal welfare along with the genetic selection for economically important production traits.

**Abstract:**

Animal health and welfare are monitored during meat inspection in many slaughter plants around the world. Carcasses are examined by meat inspectors and remarks are made with respect to different diseases, injuries, and other abnormalities. This is a valuable data resource for disease prevention and enhancing animal welfare, but it is rarely used for this purpose. Records on carcass remarks on 140,375 finisher pigs were analyzed to investigate the possibility of genetic selection to reduce the risk of the most prevalent diseases and indicators of suboptimal animal welfare. As part of this, effects of some non-genetic factors such as differences between farms, sexes, and growth rates were also examined. The most frequent remarks were pneumonia (15.4%), joint disorders (9.8%), pleuritis (4.7%), pericarditis (2.3%), and liver lesions (2.2%). Joint disorders were more frequent in boars than in gilts. There were also significant differences between farms. Pedigree records were available for 142,324 pigs from 14 farms and were used for genetic analysis. Heritability estimates for pneumonia, pleuritis, pericarditis, liver lesions, and joint disorders were 0.10, 0.09, 0.14, 0.24, and 0.17 on the liability scale, respectively, suggesting the existence of substantial genetic variation. This was further confirmed though genome wide associations using deregressed breeding values as phenotypes. The genetic correlations between these remarks and finishing traits were small but mostly negative, suggesting the possibility of enhancing pig health and welfare simultaneously with genetic improvement in finishing traits. A selection index based on the breeding values for these traits and their economic values was developed. This index is used to enhance animal welfare in pig farms.

## 1. Introduction

Meat inspection is routinely carried out in many slaughter plants around the world. In many countries, it is regulated by local government agencies such as the Canadian Food Inspection Agency (CFIA) in Canada, the Food Safety and Inspection Service (FSIS) in USA, and the European Food Safety Agency (EFSA) for European countries. The primary purpose is to check carcasses for safety of meat as a food for human consumption. Carcasses are checked by trained inspectors and remarks are made for different diseases and abnormalities. Many of the slaughter plants are very large and process thousands of carcasses every day. This generates large volumes of data on various aspects of animal health and welfare. Some slaughter plants use these data to provide feedback to the farms that supply the pigs. Apart from this, the data could be very useful for taking preventive measures for diseases at the farms and enhancing animal welfare [1,2]. However, there are limited examples of using the data for this purpose.

In this investigation, data were obtained from slaughter plants in Germany. These pigs had radio-frequency identification (RFID) tags and the plants had devices to read them during the fast moving slaughter pig chains. The data could also be linked to individual performance and pedigree records. The main objective of this study was to investigate the possibility of reducing the risk of most prevalent unfavorable health and welfare indicators through genetic selection. As part of this main objective, effects of various non-genetic factors were also examined to help in farm management.

## 2. Materials and Methods

### 2.1. Data

The data for this study consisted of slaughter remarks on 140,375 pigs from slaughter plants in Germany from July 2011 to July 2016. The data consisted of 69 slaughter remarks. The most common remarks were pneumonia (15.4%), joint disorders (9.8%), pleuritis (4.7%), pericarditis (2.3%), and liver lesions (2.2%). These traits were analyzed in detail. A total of 27.1 (%) pigs had either one or more such remark. Therefore, an additional trait was considered as the presence or absence of any of these remarks (yes = 1 or no = 0) and denoted as ‘remark’ in this manuscript. These pigs originated from 13 farms in Germany and the Netherlands. Pedigree records for five generations and records on finishing and carcass quality trait were available on 142,324 pigs from these farms and were used for genetic analysis. All these pigs were progeny of Pietrain sires and Large White x Landrace (F1) dams.

### 2.2. Slaughter Remarks Analyzed

A brief description of the slaughter remarks analyzed is given below.

Pneumonia: Is an inflammation of the tissues of the lungs. It mainly results from the response of the animal to an infectious agent, either a virus or bacteria, or in most cases both. Pneumonia was recorded at three different levels according to the severity of the lesions. For the purpose of this analysis, three classes of pneumonia were merged and pneumonia was scored as 1 present when observed regardless of intensity and 0 when absent.

Pleuritis: Also known as pleurisy, it is the inflammation of the tissue layers (pleura) lining the lungs and inner chest wall. It causes discomfort to pigs while breathing. Carcasses from affected pigs typically require stripping and removal of the inflamed pleural membrane from the thoracic cavity. In more severe cases, extensive trimming of the chest wall becomes necessary. This involves additional costs to the processor in terms of lost carcass weight, compromised carcass grading, and requirement of extra time in trimming affected areas. Severity of pleuritis was not recorded, but a remark was made as 1 when it was observed. 

Pericarditis: Pericarditis is inflammation of the pericardium, the fibrous sac surrounding the heart. It holds the heart in place and helps it work. There is a small amount of fluid between two layers of the pericardium that keeps the layers separate and avoids friction between them. Pericarditis causes chest pain to the pig. The affected carcasses require trimming that causes economic losses to the processor. This was also recorded as 0 or 1, like other remarks.

Liver lesions: Liver lesions are mainly caused by parasitic infections. White spots, also called milk spots, are observed in the liver of affected pigs. A liver was scored as 1 when more than five white spots were observed. 

Joint disorders: This included bursitis and other disorders leading to inflammation of joints. The bursa is a sac filled with lubricating fluid, located between bone, muscle, tendons, and skin to decrease rubbing, friction, and irritation. Bursitis is the inflammation or irritation of the bursa. Bursitis causes pain and difficulty in walking for the animal. Therefore, it indicates poor animal welfare. If a joint disorder was observed in a carcass, it was recorded as 1. 

Remark: In addition to remarks on the individual traits above, a general variable whether any of the remarks was recorded (yes/no) was also analyzed. It was considered as 1, when any remarks were observed. 

### 2.3. Statistical Analysis

The data were analyzed in two steps. In the first step, effects of non-genetic factors were studied using a generalized linear model in PROC GLM of SAS. The model for each trait included fixed effects of the farm of origin, sex of the pig, and date of slaughter.

In the second step, data were analyzed with a sire model using ASReml 4.0 [3]. In addition to above fixed effects, this model also included random effects of the sire and litter of the dam. It was assumed that, although the traits are observed and recorded on a binary scale (0 and 1), they follow an underlying normal distribution with a threshold [4]. Hence, the data were analyzed on a liability scale using log link function [3]. After estimation of breeding values, we applied the methodology described by Garrick et al. [5] to obtain deregressed breeding values, which is a more suitable response variable in genome-wide association studies than estimated breeding values, because of removal of parental information and removal of regression to the mean in estimated breeding values. 

A genome wide association study (GWAS) was conducted using the GCTA software developed by [6]. In the GWAS, we used the genotypes of the 597 sires and their deregressed breeding values based on the finishing offspring evaluated in the slaughter plants. These 597 sires were a subset from a larger dataset that contained 11,489 genotyped animals from the same breed. All these animals were genotyped using either the llumina Porcine SNP60 Beadchip or the GeneSeek Customer 80 K Beadchip (80 K). Additionally, 209 boars from this group were also genotyped using the Axiom porcine 660 K array and were used as the reference population in the imputation of all 11,489 genotyped with the 60 K or 80 K chips towards the 660 K. The boars genotyped with the 660 K were selected because they were frequently used AI boars and were close relatives to the remaining genotyped animals. Imputation to 660 K was performed using FImpute software version 2.2 [7] after quality control of the genotypes. The quality control excluded SNPs with a call rate lower than 0.95, a minor allele frequency lower than 0.01, and SNPs that deviated significantly from Hardy–Weinberg equilibrium (χ^2^ > 600). Unmapped SNPs and SNPs located on the sex chromosomes were also excluded. Positions of the SNPs were based on the Sscrofa10.2 assembly of the reference genome. All genotyped animals that had a frequency of missing genotypes below the threshold of 0.05 were excluded. After quality control and imputation procedures, 446,117 SNPs were used in GWAS. An association between SNPs and phenotypes was considered significant when the probability was less than 0.000001 (−log_10_ (*p*) > 6) [8].

## 3. Results

### 3.1. Prevalence of Health and Welfare Disorders in Different Farms

In the initial analysis, it was observed that there were differences between farms with respect to the incidences of the slaughter remarks observed at the slaughter plant. The remarks could be affected by the sex of the animal and by day to day differences due to handling of pigs at the farm plant and scoring by different inspectors. Therefore, least-squares means of the slaughter remarks were estimated using the generalized linear model accounting for these differences. The least-squares means for the different farms are given in Table 1. The differences between farms were highly significant (*p* < 0.001) for each of the traits. There was very high incidence of pneumonia in farms B, C, and J at 59.5, 59.1, and 57.3 percent, respectively. The pigs from these farms were slaughtered between January 2014 and June 2016. The incidence was higher than average during winter but also during summer months, indicating some unfavorable management conditions at these farms.

### 3.2. Differences between Sexes

**Figure 1:**
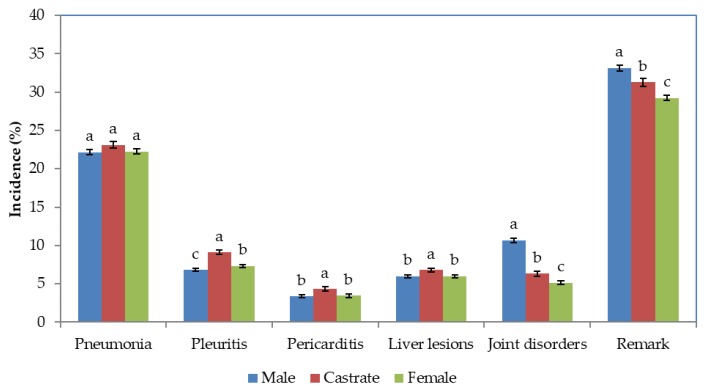
Least-squares means and standard errors of Incidences (%) of different remarks in different sexes. The standard errors are shown by vertical lines. Bars which do not share a common letter superscript were significantly different. There were significant (*p* < 0.001) differences sexes denoted by different letters.

The effects of sex was significant (*p* < 0.001) for all traits except pneumonia. Least-squares means suggest that the incidence of pneumonia was quite similar among the three sexes (Figure 1). 

However, castrates had relatively higher incidence of pleuritis, pericarditis, and liver lesions compared to males and females. The joint disorders were highest in males, followed by castrates and females. Similar pattern was observed with respect to presence or absence of any of the remarks, mainly due to the pattern of joint disorders among the sexes. 

### 3.3. Slaughter Remarks and Growth

**Figure 2:**
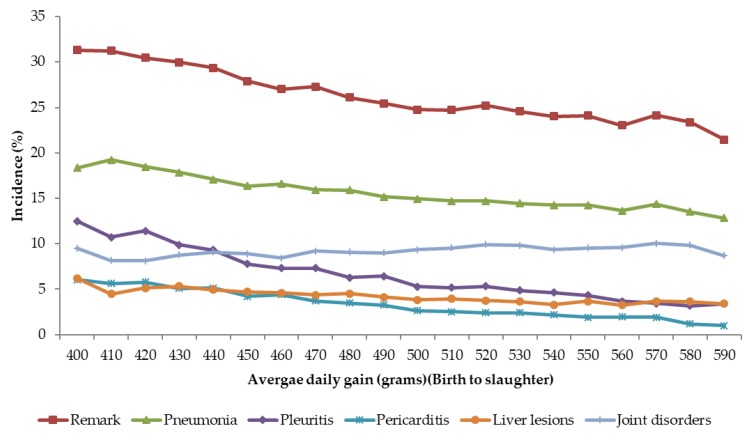
Relationship between growth and incidences (%) of different remarks.

Relationship between slaughter remarks and growth is shown in Figure 2. The average daily gain from birth to slaughter was divided into classes of 10 g/day. The incidences in each class are presented as least-squares means accounting for differences in farm, sex, and slaughter date. In general, there is tendency for the lower remarks with increase in growth rate, except for joint disorders that show a slightly increasing trend. These results show that a faster growth rate is not necessarily associated with unfavorable effects on welfare. In fact, fast growing pigs have fewer incidences of the diseases, therefore leading to less pain and discomfort to the animals.

**Figure 3:**
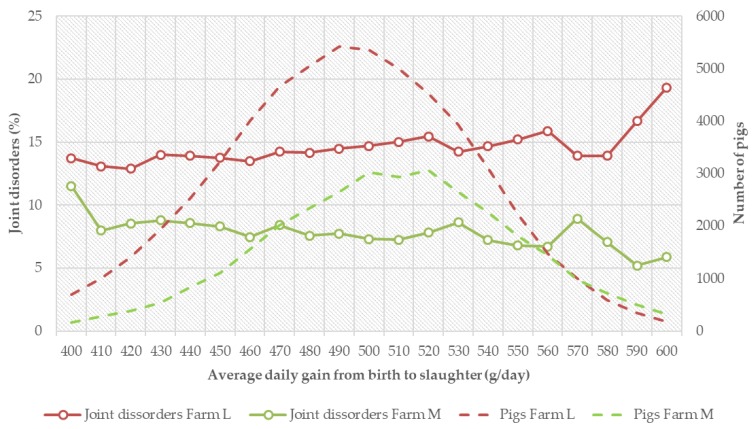
Relationship between growth from birth to slaughter in different classes and incidences of joint disorders (%) in two farms. The broken lines show distributions of pigs with respect to growth rate in the farms.

The relationship between growth and joint disorders differed among the farms. In some farms, there was a favorable trend as the pigs with higher growth rate had lower incidences while, in some other farms, the relationship was reversed. Examples of the patterns in two largest farms are given in Figure 3. In farm L, there was an increase in incidence of joint disorders in pigs with higher growth rate while, in farm M, there was a tendency for lower joint disorders. The number of pigs in each growth rate class is also shown in Figure 3, on the secondary *y*-axis. There was a higher frequency of pigs with lower growth rate in farm L than in farm M. The average daily gain from birth to slaughter in farm L was also lower (490 g/day) than in Farm M (507 g/day). The farm L with slower growth rate higher average incidence for joint disorder (13.1%) than farm M (6.1%) which had a faster growth rate. This is the reverse order of the trend in Figure 2. These results suggest that the relationship between growth rate and joint disorders is very much dependent on the farm management. Farms with better management and higher average growth rate could actually have lower incidences of joint disorders. In this study, about 42% of the pigs were from Farm L that had lower growth rate and higher incidence of joints. That might have contributed to the overall slightly unfavorable association of growth rate and joint disorders (Figure 2). 

### 3.4. Estimates of Variance Components and Heritability

The estimates of variances due to genetic and common litter effects are given in Table 2. As this analysis was conducted on a liability scale using log link function, the residual variance is considered to be a fixed value of 3.29. The genetic variance was highest for liver lesions and joint disorders followed by pneumonia, pleuritis, and pericarditis. However, the common litter effect was lowest for joint disorders compared to other traits, explaining less than 1% of the genetic variance. This variance accounted for 3.7, 5.7, 8.1, and 5.8 percent of the total phenotypic variance in pneumonia, pleuritis, pericarditis, and liver lesions, respectively. This suggests higher predisposition to these traits by common maternal environment. The heritability estimates for pneumonia, pleuritis, pericarditis, liver lesions, and joint disorders were 0.10, 0.09, 0.14, 0.24, and 0.17, respectively. These estimates are not high enough to deliver dramatic improvements, but are high enough to drive some improvement. 

### 3.5. Associated Genomic Regions

**Figure 4:**
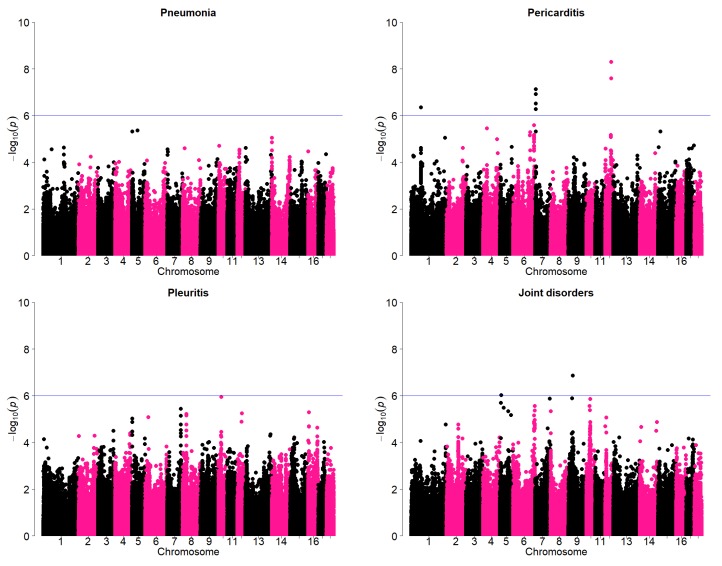
Manhattan plots showing genome-wide association results for pneumonia, pleuritis, pericarditis, and joint disorders. SNPs are plotted on the *x*-axis according to their position on each chromosome against statistical association with these traits on the *y*-axis. The horizontal line indicates genome-wide significance (*p* < 1.0^−6^).

Results of the genome wide association study (GWAS) to further verify the existence of genetic variation for these traits are given in Figure 4. Significant SNPS above the threshold were observed for pericarditis and joint disorders. A majority of the most significant regions appear to be on the tail ends of the chromosomes. There are some concerns that this could be an artifact as annotations towards the tail ends of the chromosomes may not be very accurate. Therefore, some examples of the SNP effect were examined in detail which suggests that the SNPs actually had significant effects. An example is given in Table 3. In this example, the most significant SNP on chromosome 7 (AX-116309387) for pericarditis is considered. The frequency of the most favorable genotype for this SNP was lowest among the sires. The average incidence of pericarditis in was lowest in the most favorable genotype (1.9%) compared to intermediate (2.5%) and the unfavorable genotype (2.6%). These differences among the least-squares means of the most favorable and other genotypes were actually statistically significant (*p* < 0.001). Pericarditis could lead to pain and in death of adult sows. In a study of the most important causes of sow mortality, heart failure was considered to be most important cause [9]. Although the incidence of pericarditis is not very high, the results suggest availability of genomic regions that could have relatively large effect.

### 3.6. Phenotypic and Genetic Correlations Among Slaughter Remarks

In general, there were very low phenotypic correlations among the slaughter remarks (Table 4). Especially the correlations of joint disorders with other traits were close to zero. The highest phenotypic correlation was between pleuritis and pericarditis. This was followed by correlation between pleuritis and pneumonia. This is expected, as inflammation due to infection of heart and lungs could also lead to inflammation of the pleura surrounds the heart and lungs. The relative magnitudes of correlations between the individual remarks and the computed variable remark (yes or no) mainly represent their relative contributions to this computed variable. 

The genetic correlations between the slaughter remarks were of similar magnitude or even higher. However, they were associated with larger standard errors. The correlations among slaughter remarks were close to zero and non-significant for most of the traits. It was highest between pleuritis and pericarditis and even larger than the phenotypic correlation, suggesting similar genetic predisposition for both the traits. However, the genetic correlation between pneumonia and pleuritis was lower than phenotypic correlation and not significantly different from zero. This suggests that the phenotypic association was mainly due to the transmission of infection between the lungs and pleura. The genetic correlation between pericarditis and liver lesions was higher than the respective phenotypic correlation but still lower than the correlation between pericarditis and pleuritis. The genetic correlations of joint disorders with pneumonia, pleuritis, and pericarditis were low and non-significant (*p* > 0.05), except for the correlation with liver lesions. 

### 3.7. Genetic Correlations with Production Traits

Estimates of genetic correlations with different production traits are given in Table 5. These include traits of finishing and carcass quality. A majority of these correlations were quite low and non-significant. In general, these correlations are not expected to be very high as they reflect lesions observed at slaughter mainly due to diseases that are more chronic in nature. Nevertheless, these lesions are indicative of long term pain and suffering, therefore indicating poor animal welfare. The correlations were in favorable direction for pneumonia, pleuritis, and pericarditis, and unfavorable for liver lesions. The genetic correlations of joint disorders with average daily gain from birth to slaughter and with carcass backfat were slightly larger, suggesting that joint disorders are associated with adverse effects on these traits. The genetic correlation of backfat with joint disorders and remark were low but unfavorable to selection for reducing backfat. These correlations actually suggest that leaner pigs could have higher joint disorders and overall higher remarks. The correlation between growth and remark was in favorable direction, suggesting pigs with faster growth tend to have lower risks associated with these indicators of suboptimal health and welfare.

## 4. Discussion

The results suggest the existence of genetic variation for slaughter remarks and opportunities to reduce their prevalence through genetic selection. Therefore, a selection index was developed for identification of genetically superior boars whose progeny are expected to have a lower risk of unfavorable slaughter remarks. This index was calculated as sum of product of the breeding values with their respective economic weights. It was quite difficult to get the most current estimates of gains and losses to producers and slaughter plants for individual slaughter remarks. The information is either not available or it is not shared. Hence, the economic weights were approximated with available information. The economic values for the slaughter remarks were based on [10] and adapted to the incidences of the slaughter remarks in current study and current prices. This index is used to identify boars that can be used by producers to enhance animal welfare in their pig farms. 

In this study, it was possible to obtain data on individual pigs from the slaughter plants. This is not very common, particularly in North America, and in many other regions as the slaughtered pigs do not have individual identifiers. In those plants, the observations are recorded on a group, e.g., based on slap tattoo of a batch of pigs. However, with increasing awareness in food safety and animal welfare, many slaughter plants are putting them in place. This will allow for easier linkage to farm records and more analysis on various aspects of disease prevention and genetics.

There are several concerns regarding accuracy and usefulness of data from slaughter plants as the data are recorded in fast moving chains by different inspectors in different plants. The concerns include a lack of ability of the meat inspector to detect affected carcasses in the fast moving slaughter chain, lack of standardization among the inspectors and conditions in the slaughter plant, and that only apparently healthy pigs are sent to slaughter [11].

All these factors were also carefully evaluated during his study by visiting the slaughter plants, observing the inspection process and through discussion with the staff. Another issue is the relationship of the observations at the slaughter plants and on the actual farm level. In Danish finisher pigs, ref. [12] observed moderate correlations between the routine meat inspection data and data from systematic health monitoring system for pleuritic and lung lesions but poor for pericarditis. The coefficients of determination (R^2^) were 0.67 for pleuritis and 0.40 for lung lesions and 0.16 for pericarditis. Similarly, ref. [13] observed low sensitivity (probability of detecting conditions actually present in the diseased group) and specificity (probability of correctly identifying healthy carcasses in a healthy group) for heart disorder in a comparison of routine abattoir inspections for heart disorders in a study that compared two groups of observers (regular meat inspectors and two veterinary researchers). 

Therefore, efforts were made to record the identity of the inspector to account for the differences among their remarks in the statistical model. However, it was not possible due to several factors including the regulations relating to the data recording by government officials. Therefore, an attempt was made to partly account for these differences by including the effects of slaughter dates. It is expected that these inaccuracies will have minimal effect on estimates of genetic parameters due to the large data size and the genetic evaluation models used. In a similarly large study of the Food Standards Agency (FSA), data from over 19 million pigs from three abattoirs in Great Britain were gathered and compared with data from a targeted surveillance system of slaughtered pigs (BHPS) and laboratory diagnostic scanning surveillance (FarmFile), [1] also concluded that there is potential to use FSA data as a component of a surveillance system to monitor temporal trends and regional differences at population level, in spite of the aforementioned difficulties.

Management plays a crucial role in the occurrence of meat inspection remarks. Management is the first and major factor to be addressed. That is one of the main reasons for differences between farms. At the same time, there is meaningful genetic variation. In fact, some genetic variation should be expected as some full sib or half sib families have better joints, lungs, hearts, livers, etc. than others that make them less liable to diseases related to these organs. Genetic selection for the better families or genetic lines can reduce the incidence of infections and, with that, reduce the chance that other animals are infected. However, genetic selection for more robust animals should not reduce the focus on management improvements.

To our knowledge, this is the first example of using meat inspection data for genetic selection to enhance animal welfare in pigs. A system has been put in place to obtain these data by direct links with the organizations responsible. This is allowing routine genetic evaluations and selection of pigs using the welfare index to reduce the risk of unfavorable slaughter remarks. In this way, this is also an example of cooperation along the pork value chain, from processing back to production and genetics, as a concerted effort of the value chain partners is required to enhance animal welfare.

## Figures and Tables

**Table 1:** Least-squares means of incidence (%) of slaughter remarks in different farms.

Farm	Pigs	Pneumonia	Pleuritis	Pericarditis	Liver Lesions	Joint Disorders	Remark
A	18,665	10.1	4.9	1.3	2.5	9.4	22.1
B	2319	59.5	2.4	10.0	3.5	1.6	61.0
C	1630	57.3	2.7	2.6	3.6	2.6	59.9
D	6664	14.9	20.5	7.8	4.4	7.2	35.0
E	883	8.6	11.7	7.3	11.9	8.1	15.5
F	7212	8.8	2.1	10.7	2.0	7.7	14.5
G	3981	7.4	2.3	3.6	2.4	9.0	12.7
H	4287	16.9	20.5	11.4	3.5	5.0	26.9
I	266	9.1	8.9	7.3	3.7	12.0	20.4
J	1343	59.1	7.0	4.6	3.0	4.8	64.5
K	1722	10.2	9.0	11.1	2.5	9.1	20.8
L	59,079	17.3	4.8	1.9	3.1	13.1	31.3
M	32,324	13.5	4.1	1.8	2.2	6.1	21.3
All	140,375	15.4	4.7	2.3	2.2	9.8	27.1

**Table 2:** Variance component estimates and heritability.

Component	Pneumonia	Pleuritis	Pericarditis	Liver Lesions	Joint Disorders	Remark
Genetic	0.368 (0.033)	0.336 (0.057)	0.562 (0.098)	0.939 (0.134)	0.626 (0.046)	0.360 (0.027)
Common litter	0.134 (0.016)	0.208 (0.035)	0.314 (0.062)	0.231 (0.066)	0.035 (0.018)	0.098 (0.011)
Residual	3.290 (0.000)	3.290 (0.000)	3.290 (0.000)	3.290 (0.000)	3.290 (0.000)	3.290 (0.000)
Phenotypic	3.609 (0.019)	3.665 (0.036)	3.885 (0.061)	3.991 (0.072)	3.638 (0.024)	3.568 (0.015)
Heritability	0.10 (0.01)	0.09 (0.02)	0.14 (0.02)	0.24 (0.03)	0.17 (0.01)	0.10 (0.01)

**Table 3:** Effect of the genotype of the sire with respect to most significant SNP on chromosome 7 on pericarditis in their progeny.

Genotypes	Sires	Progeny	Lsmean	S.E.	Pr > |t|
0	48	4909	0.019 ^a^	0.003	<0.0001
1	240	24,195	0.025 ^b^	0.003	<0.0001
2	309	30,012	0.029 ^b^	0.003	<0.0001
Total	597	59,116			

Values with different superscripts within this column are significantly different (P < 0.05)。

**Table 4:** Estimates of genetic and phenotypic corrections among slaughter remarks. Phenotypic correlations above diagonal, genetic correlations below diagonal.

Trait	Pneumonia	Pleuritis	Pericarditis	Liver Lesions	Joint Disorders	Remark
Pneumonia		0.09 ** (0.003)	0.11 ** (0.003)	0.03 ** (0.003)	0.00^NS^ (0.003)	0.70 ** (0.002)
Pleuritis	0.05 ^NS^ (0.027)		0.30 ** (0.003)	0.05 ** (0.003)	−0.02 ** (0.003)	0.37 ** (0.003)
Pericarditis	0.11 ** (0.027)	0.43 ** (0.025)		0.05 ** (0.003)	0.00 ^NS^ (0.003)	0.25 ** (0.003)
Liver lesions	0.04 ^NS^ (0.028)	0.05 ^NS^ (0.027)	0.12 ** (0.027)		−0.02 ** (0.003)	0.02 ** (0.003)
Joint disorders	0.02 ^NS^ (0.028)	0.05 ^NS^ (0.028)	0.04 ^NS^ (0.028)	0.11 ** (0.027)		0.52 ** (0.003)
Remark	0.69 ** (0.020)	0.34 ** (0.026)	0.22 ** (0.027)	0.08 ** (0.027)	0.57 ** (0.023)	

NS = *p* > 0.05, * = *p* < 0.05, ** = *p* < 0.01.

**Table 5:** Genetic correlations with finishing and carcass quality traits.

Trait	Pneumonia	Pleuritis	Pericarditis	Liver Lesions	Joint Disorders	Remark
Average daily gain	−0.07 * (0.027)	−0.10 ** (0.027)	−0.05 (0.028)	0.02 (0.028)	0.09 ** (0.027)	−0.07 * (0.027)
Backfat	−0.03 (0.028)	0.02 (0.028)	0.02 (0.028)	−0.02 (0.028)	−0.11 ** (0.027)	−0.06 * (0.027)
Loin depth	−0.02 (0.028)	−0.02 (0.028)	−0.02 (0.028)	0.01 (0.028)	0.01 (0.028)	−0.03 (0.028)
Ham weight	−0.01 (0.028)	−0.03 (0.028)	−0.01 (0.028)	0.03 (0.028)	0.03 (0.028)	−0.02 (0.028)
Loin weight	−0.01 (0.028)	−0.02 (0.028)	−0.02 (0.028)	0.03 (0.028)	0.03 (0.028)	−0.01 (0.028)
Shoulder weight	−0.01 (0.028)	−0.02 (0.028)	−0.02 (0.028)	0.04 (0.028)	0.04 (0.028)	−0.01 (0.028)
Belly weight	−0.01 (0.028)	−0.02 (0.028)	0.01 (0.028)	0.05 (0.027)	0.01 (0.028)	−0.02 (0.028)

NS = *p* > 0.05, * = *p* < 0.05, ** = *p* < 0.01.
